# COVID-19 Vaccination Knowledge and Attitude Among the General Population in Jeddah, Saudi Arabia

**DOI:** 10.7759/cureus.42951

**Published:** 2023-08-04

**Authors:** Adeeb Munshi, Ahmad Alhouthali, Enas Munshi, Mohamed K Mujalled, Lama Alqalayta, Hala Zahed, Nawal Almahmoudi, Laila A Alghubayshi, Mariya Bahashwan, Abdulhakeem Althaqafi

**Affiliations:** 1 College of Medicine, King Saud Bin Abdulaziz University for Health Sciences, Jeddah, SAU; 2 Infectious Diseases, King Abdullah International Medical Research Center, Jeddah, SAU; 3 Medicine/Infectious Diseases, King Abdulaziz Medical City, Jeddah, SAU; 4 Internal Medicine, King Abdulaziz Medical City, Jeddah, SAU; 5 Infectious Diseases, King Abdulaziz University Hospital, Jeddah, SAU

**Keywords:** attitude, knowledge, vaccination, saudi arabia, general population

## Abstract

Background

The population’s hesitancy to get the coronavirus disease 2019 (COVID-19) vaccination may pose a risk to public health worldwide. However, the causes and contributors to vaccine hesitancy in the Saudi people need to be understood. This study aimed to assess the knowledge and attitudes toward COVID-19 vaccination among the general population in Jeddah, Saudi Arabia.

Methodology

A cross-sectional study was conducted using an online structured questionnaire titled “Knowledge and attitude toward COVID-19 vaccination.” The questionnaire collected demographic characteristics, knowledge, and attitudes toward COVID-19 vaccination among the general population in Jeddah, Saudi Arabia. The questionnaire was distributed through links on various sites, including Twitter and WhatsApp.

Results

A total of 544 participants were included in the present study. Overall, 64.9% were males, and 35.1% were females. Most of the population had received three doses (83.6%) of the COVID-19 vaccine. About half of the participants had a university education (56.8%), 17.5% of respondents were postgraduates, and 14.2% had secondary education. Overall, 41.4% of the population had a low knowledge level, and 58.5% had a low attitude toward COVID-19 vaccination. Receiving the COVID-19 vaccine was significantly (p < 0.05) associated with the population’s knowledge and attitude toward COVID-19 vaccination. In addition, the education level substantially affected the knowledge and attitude toward COVID-19 vaccination. Additionally, knowledge of COVID-19 vaccination had a significant correlation (p < 0.05) with the attitude toward the vaccine.

Conclusions

Despite the high prevalence of the population who received the three doses of the COVID-19 vaccine in Jeddah city, the study showed insufficient knowledge and attitude among the population toward COVID-19 vaccination. However, fortunately, education plays an essential role in improving the knowledge of COVID-19 vaccination.

## Introduction

Globally, healthcare organizations have wished for a safe and efficient vaccination for the coronavirus disease 2019 (COVID-19) caused by the severe acute respiratory syndrome coronavirus-2 (SARS-CoV-2) [1,2]. The development of vaccines takes a long time and involves several potency, effectiveness, and safety checks, especially in high-risk groups, including the elderly, pregnant women, and those with comorbid conditions and immunodeficiencies [3].

The acceptance of the recently introduced vaccine is another factor to be considered, as the population’s vaccination coverage rate is crucial for the success of an immunization program. With the COVID-19 vaccine being available just nine months after the virus was discovered, the program for its release has been hastened. Although some preliminary evidence points to the safety and effectiveness of the licensed vaccines, long-term efficacy and any potential adverse effects remain unclear [4].

Understandably, medical professionals and the general public are hesitant about the new vaccine’s reception [5]. A significant anti-vaccine movement has also gained ground, and the media has been inundated with several pseudoscientific conspiracy theories. These factors make vaccine reluctance one of the biggest obstacles to the COVID-19 vaccination program [6].

In most countries, vaccination against COVID-19 is optional; thus, before the vaccination program is implemented, it is crucial to establish the current perceptions of the local population. The worldwide COVID-19 pandemic brought the scientific community together to better comprehend the SARS-CoV-2 immune response and pathogenic mechanism. As a result, some vaccines succeeded and passed phase 3 clinical testing after a year of joint work [7]. After the COVID-19 vaccines were authorized in late 2020, vaccine manufacturers began shipping doses to various nations in early 2021. To prioritize the immunization of the elderly, individuals with chronic conditions, and medical professionals, PfizerBioNTech and AstraZeneca devised a system for online vaccination booking. These vaccines, in addition to Moderna and Johnson & Johnson (Janssen) vaccines, were licensed in Saudi Arabia [8]. According to the authorities, all Saudi and foreign inhabitants are eligible for immunization. However, vaccine hesitancy is a hotly debated public issue that may impact the government’s immunization plan to halt the epidemic [9].

According to several studies, the population’s hesitancy to get the COVID-19 vaccination may pose a risk to public health worldwide [10,11]. However, the causes and contributors to vaccination hesitancy in the Saudi population need to be understood. The causes of this hesitation might range from false information about the effectiveness and safety of the vaccines to misunderstandings about the necessity of immunization. In addition, sociodemographic factors and culture may impact vaccine reluctance [12-14]. Therefore, this study aims to assess the knowledge and attitudes toward COVID-19 vaccination among the general population in Jeddah, Saudi Arabia.

## Materials and methods

Study design

This study used a descriptive survey design. Researchers can use correlation survey data to predict future trends and explain the characteristics of a population or variations among the general population of Jeddah. Further, the reliability of this design in preserving respondents’ anonymity, which motivates them to provide truthful responses, makes it appropriate for this study.

Study population

The questionnaire was distributed among the general population in Jeddah, Saudi Arabia. Consent was taken from responders before proceeding with the questionnaire. Responders were informed of the study purpose and background on the first page of the questionnaire. Respondents were informed that they could withdraw without providing a reason at any time and that their information and opinions would remain anonymous.

Study instrument

A structured questionnaire called “Knowledge and Attitude of COVID-19 Vaccine Among the General Population in Jeddah” was used in the study, and several instrument components were self-developed. The attitude scale was adopted from Paul et al. [15] with Cronbach’s alpha values of 0.91-0.94, and the knowledge scale was derived from the Centers for Disease Control and Prevention [16].

The questionnaire consisted of three parts. The first part concerned demographic information such as sex, age, education level, and vaccination status against COVID-19. The second part included six questions assessing the responder’s knowledge of the COVID-19 vaccine. The third part included four questions assessing the responder’s attitude toward the COVID-19 vaccine. Brislin’s back-translation model was used to translate the questionnaire into Arabic [17]. Two infection control and public health experts reviewed the questionnaire for validity.

Data collection

The participants were recruited using a chain-referral sampling technique. The questionnaire was distributed through links on various sites, including Twitter and WhatsApp. Responders were asked to distribute the questionnaire among their family, friends, and coworkers.

Statistical analysis

Data were simultaneously entered into a preform and updated. It was entered into Microsoft Excel (MS Office 2010) (Microsoft® Corp., Redmond, WA, USA). SPSS software version 22.0 (IBM Corp., Armonk, NY, USA) was used for data analysis. Descriptive analysis included the computation of frequencies and percentages. Numbers were used to represent categorical variables, whereas quantitative variables were described using mean ± standard deviation (SD).

The Shapiro-Wilk test was used to determine if the variable distribution was normal. When comparing parametric variables between study groups, one-way analysis was used, and when comparing non-parametric variables, the Kruskal-Wallis test was used. An analysis of qualitative variables was conducted using the chi-square test. A p-value of less than 0.05 was considered significant.

Responses were rated on a four-point scale ranging from 1, “strongly agree,” to 4, “strongly disagree.” The mean attitude score was calculated by multiplying the number of points on the scale by the number of responses divided by the total number of respondents.

Ethical consideration

The study approval was obtained from the Institutional Review Board of King Abdullah International Medical Research Center (approval number: NRJ22J/011/01).

## Results

A total of 544 participants were included in the present study. Table 1 shows that most participants were males (64.9%), and 35.1% were females. Most of the population had received three doses (83.6%) of the COVID-19 vaccine. About half of the participants had a university education (56.8%), 17.5% of respondents were postgraduates, and 14.2% had a secondary education.

**Table 1:** Demographic information of the respondents.

Variable	Number	Percentage (%)
Age (years)		
18–24	135	24.8%
25–30	88	16.2%
31–35	66	12.1%
36–40	62	11.4%
41–45	53	9.7%
>45	140	25.7%
Gender		
Male	353	64.9%
Female	191	35.1%
Have you received the novel COVID-19 vaccine?		
No	5	0.9%
Yes one dose	2	0.4%
Yes two doses	60	11%
Yes three doses	455	83.6%
Yes four doses	22	4%
Education level		
Intermediate	9	1.7%
Diploma	54	9.9%
Secondary	77	14.2%
University	309	56.8%
Postgraduate	95	17.5%

Table 2 shows the knowledge of the respondents about the approved COVID-19 vaccines, including Pfizer-BioNTech (93.9%), AstraZeneca-Oxford (66.9%), Moderna (43.8%), and Johnson & Johnson’s Janssen (14%). The majority of respondents (70.4%) answered “No” when they were asked about the ability of the new COVID-19 vaccine to change the composition of genes inside the body. Half the study population (50%) thought the new vaccine could protect them from COVID-19. Overall, 21.7% of respondents felt they do not need the COVID-19 vaccine if they had contracted the disease before. Most participants (95.6%) thought that after taking the vaccine against COVID-19, they could become infected with the emerging COVID-19. Additionally, 35.5% of participants believed that the vaccine against COVID-19 might give them the emerging COVID-19.

**Table 2:** Knowledge of the COVID-19 vaccine among the study participants.

Knowledge of COVID-19 vaccination	Yes	No
Approved COVID-19 vaccines		
AstraZeneca–Oxford COVID-19 vaccine	364 (66.9%)	180 (33.1%)
Pfizer–BioNTech COVID-19 vaccine	511 (93.9%)	33 (6.1%)
Johnson & Johnson’s Janssen COVID-19 vaccine	76 (14%)	468 (86%)
Moderna COVID-19 vaccine	238 (43.8%)	306 (56.3%)
The COVID-19 vaccine can change the composition of genes inside my body	161 (29.6%)	383 (70.4%)
The COVID-19 vaccine can protect me from COVID-19	272 (50%)	272 (50%)
I do not need the COVID-19 vaccine if I have had the disease before	118 (21.7%)	426 (78.3%)
After taking the COVID-19 vaccine, I can become infected with COVID-19	520 (95.6%)	24 (4.4%)
The COVID-19 vaccine may infect me with the COVID-19 virus	193 (35.5%)	351 (64.5%)

Table 3 shows the attitude toward the COVID-19 vaccine among the study participants. Overall, 32.7% of respondents strongly disagreed that they do not trust the vaccine’s benefit against COVID-19. Moreover, 36.2% of participants agreed that they have concerns about the unexpected side effects of the vaccine. In addition, 28.3% of respondents strongly agreed that they were concerned about exploitative trading from manufacturers. Furthermore, 24.3 of respondents preferred natural immunity without vaccinations.

**Table 3:** The attitude toward the COVID-19 vaccine among the study participants.

The attitude toward the COVID-19 vaccine among the study participants	Strongly disagree	Disagree	Agree	Strongly agree	Mean	SD
I do not trust the benefit of the vaccine against the coronavirus	178 (32.7%)	215 (39.5%)	95 (17.5%)	56 (10.3%)	2.05	0.96
I have concerns about the unexpected side effects of the vaccine	74 (13.6%)	151 (27.8%)	197 (36.2%)	122 (22.4%)	2.67	0.97
I have a concern about exploitative trading from manufacturers	57 (10.5%)	144 (26.5%)	189 (34.7%)	154 (28.3%)	2.81	0.97
I prefer natural immunity without vaccinations	106 (19.5%)	187 (34.4%)	132 (24.3%)	119 (21.9%)	2.49	1.04

Table 4 shows that 41.4% of the population had a low knowledge level about COVID-19 vaccination. In addition, 58.5% of participants had a low attitude about COVID-19 vaccination.

**Table 4:** Overall knowledge and attitude levels about COVID-19 vaccination.

Variable	Number (%)
Knowledge level	
High	319 (58.6%)
Low	225 (41.4%)
Attitude level	
High	226 (41.5%)
Low	318 (58.5%)

Table 5 shows receiving the novel COVID-19 vaccine was significantly (p < 0.05) associated with the population’s knowledge and attitude toward COVID-19 vaccination. In addition, the education level substantially affected the knowledge and attitude toward COVID-19 vaccination.

**Table 5:** Correlation of knowledge and attitude toward the COVID-19 vaccination scores with other variables. P: p-value. The significance level was set at p-values <0.05.

Variable		Knowledge	Attitude
Age	P	0.224	0.259
Have you received the novel COVID-19 vaccine?	P	0.000	0.000
Education level	P	0.022	0.010

Table 6 shows that receiving the novel COVID-19 vaccine was significantly (p < 0.05) associated with the knowledge of COVID-19 vaccination; 87.1% of the population with high knowledge of COVID-19 vaccination had taken three doses of the COVID-19 vaccine. In addition, the education level significantly (p = 0.040) affected the knowledge level of COVID-19 vaccination. Participants with a university education level (57.4%) had a high knowledge level. Additionally, knowledge of COVID-19 vaccination had a significant correlation (p < 0.05) with the attitude toward the vaccine.

**Table 6:** Relations of the knowledge level of COVID-19 vaccination with the basic characteristics. The significance level was set at p-values <0.05.

	Knowledge level		
	High	Low	P-value
Age (years)			
18–24	83 (26%)	52 (23.1%)	0.401
25–30	55 (17.2%)	33 (14.7%)	
31–35	37 (11.6%)	29 (12.9%)	
36–40	37 (11.6%)	25 (11.1%)	
41–45	24 (7.5%)	29 (12.9%)	
>45	83 (26%)	57 (25.3%)	
Gender			
Male	208 (65.2%)	145 (64.4%)	0.855
Female	111 (34.8%)	80 (35.6%)	
Have you received the novel COVID-19 vaccine?			
No	0 (0)	5 (2.2%)	0.000
Yes one dose	1 (0.3%)	1 (0.4%)	
Yes two doses	22 (6.9%)	38 (16.9%)	
Yes three doses	278 (87.1%)	177 (78.7%)	
Yes four doses	18 (5.6%)	4 (1.8%)	
Education level			
Intermediate	2 (0.6%)	7 (3.1%)	0.040
Diploma	31 (9.7%)	23 (10.2%)	
Secondary	39 (12.2%)	38 (16.9%)	
University	183 (57.4%)	126 (56%)	
Postgraduate	64 (20.1%)	31 (13.8%)	
Attitude level			
High	73 (22.9%)	153 (68%)	0.000
Low	246 (77.1%)	72 (32%)	

Receiving the novel COVID-19 vaccine was significantly (p = 0.004) associated with the population’s attitude toward COVID-19 vaccination. Additionally, knowledge of COVID-19 vaccination had a significant correlation (p = 0.036) with the attitude toward the vaccine.

## Discussion

The Kingdom of Saudi Arabia (KSA) is home to 34.81 million people. Since March 2020, when the first COVID-19-positive case was reported, KSA has had the second-highest number of COVID-19 infections in the Gulf region. The government has implemented several preventative steps to stop the spread of the disease, including providing free vaccinations to everyone over 12 years of age [18]. Every new medical procedure has a different rate of public acceptance. One significant problem with the populace is vaccine reluctance. Numerous elements were necessary, including the media environment, communication, and public perception. It has been noted that the information provided by these sites influences people’s attitudes and knowledge of vaccinations, impeding mass immunization campaigns [19]. A scientific method for developing solutions to problems and accomplishing the authorities’ intended goals in society is to analyze people’s knowledge and other perception issues based on facts and statistics on practices [19].

This study included 544 participants with ages ranging between 18 and older than 45 years. Most of the study population had received three doses (83.6%) of the novel COVID-19 vaccine. Most of them had knowledge about the approved COVID-19 vaccines, such as Pfizer-BioNTech, AstraZeneca-Oxford, and Moderna. In an Ethiopian study by Abebe et al. (2021), most responders (83.3%) were aware of the efficacy of the newly developed COVID-19 vaccination [20]. The percentage of males (64.9%) was higher than females (35.1%) in our study, which is different from a survey conducted by Almaghaslah et al., which reported a higher percentage of females (54.5%) than males (45.5%) [11]. Most participants (74.3%) had a university education and above, which is consistent with a study conducted in KSA, which reported 67.5% of the participants with a university education and above [11].

In this study, age, education level, and receiving the novel COVID-19 vaccine significantly affected the knowledge and attitude toward COVID-19 vaccination. Al-Marshoudi et al. (2021) reported that a history of chronic disease, source of vaccine knowledge, and education level affected the willingness to accept the vaccine [21]. Previously, a cross-sectional e-survey by Islam et al. (2021) observed that in a majority of female respondents, higher education, living nuclear families, and past vaccination usage were associated with considerably more positive attitudes toward vaccination [22].

Recently, age was found to be a significant predictor of vaccination status by Alatrany et al. (2023), with older age being linked to a higher chance of immunization. Furthermore, receiving the novel COVID-19 vaccine was significantly associated with the knowledge of COVID-19 vaccination; 87.1% of the population with high knowledge of COVID-19 vaccination had taken three doses of the COVID-19 vaccine. Additionally, knowledge about the COVID-19 vaccine is highly influenced by the level of education. Participants with a university education level (57.4%) had a higher knowledge level. Additionally, knowledge of COVID-19 vaccination significantly correlated with the attitude toward the vaccine. In Bangladesh, Rahman et al. (2022) showed that 58.13% and 64.81% of university students reported positive knowledge and attitude toward the COVID-19 vaccine, respectively [23]. Additionally, in a study by Hammour et al. (2022), with a median knowledge score of 4 out of 8, the participants had insufficient knowledge. Participants who were over 45 years of age, had undergraduate or graduate degrees, or had degrees in the medical field scored better on understanding COVID-19 vaccinations than the other participants. Higher knowledge ratings were achieved by those who showed a willingness to acquire the vaccination, registered to do so, and had already received it [24]. Chilongola et al. (2022) observed poor vaccination rates of 6.9% due to unfavorable attitudes about COVID-19 immunization despite a reasonable understanding of the disease and good preventative measures. Reluctance to vaccination appears to be significantly influenced by false information about COVID-19 [25]. In Italy, Kibi et al. (2023) indicated that demographic data, in particular, age, gender, experience with influenza vaccine, and knowledge about the disease, were significant determinants for choosing to vaccinate against COVID-19 [26].

There are many limitations of our study. One of the limitations is the use of an online survey, which could have restricted the representativeness of the study sample. Another limitation is the study’s hypothetical design, which allows the study findings to vary from real-world experience, and individual responses may contribute to information bias.

## Conclusions

This study showed that 58% of the respondents had high knowledge of the COVID-19 vaccine, while 41% of the respondents had a positive attitude toward the COVID-19 vaccine. Receiving the novel COVID-19 vaccine and education level were significantly associated with the level of knowledge and attitudes toward COVID-19 vaccination. Additionally, knowledge of COVID-19 vaccination levels significantly correlated with the attitude toward the vaccine. Therefore, despite the high prevalence of the population who received three doses of the COVID-19 vaccine in Jeddah city, this study showed insufficient knowledge and attitude among the population toward COVID-19 vaccination. But, fortunately, education played an essential role in improving the knowledge of COVID-19 vaccination.

## Appendices

**Table 7:** Demographic information.

Demographic information	Choose one
Age	
18–24	
25–30	
31–35	
36–40	
41–45	
>45	
Gender	
Male	
Female	
Have you received the novel coronavirus vaccine?	
No	
Yes one dose	
Yes two doses	
Yes three doses	
Yes four doses	
Education level	
Intermediate	
Diploma	
Secondary	
University	
Postgraduate	

**Table 8:** Knowledge of COVID-19 vaccination.

Knowledge of COVID-19 vaccination	Yes	No
Approved COVID-19 vaccines		
AstraZeneca–Oxford COVID-19 vaccine		
Pfizer–BioNTech COVID-19 vaccine		
Johnson & Johnson’s Janssen COVID-19 vaccine		
Moderna COVID-19 vaccine		
The new Corona vaccine can change the composition of genes inside my body		
The new Corona vaccine can protect me from corona disease		
I do not need the Corona vaccine if I have had the disease before		
After taking the vaccine against the coronavirus, I can become infected with the emerging corona disease		
The vaccine against the coronavirus may give me the emerging corona disease		

**Table 9:** Attitude of COVID-19 vaccination.

Attitude toward COVID-19 vaccine	Yes	No
I do not trust the benefit of the vaccine against the coronavirus		
I have concerns about the unexpected side effects of the vaccine		
I have a concern about exploitative trading from manufacturers		
I prefer natural immunity without vaccinations		

## References

[1] Chakraborty I, Maity P (2020). COVID-19 outbreak: migration, effects on society, global environment and prevention. Sci Total Environ.

[2] Shaikhain T, Al-Husayni F, Bukhari G (2021). Knowledge and attitude toward coronavirus disease 19 pandemic among Saudi Arabia population: a cross-sectional study. SAGE Open Med.

[3] World Health Organization (2020). WHO SAGE Roadmap for Prioritizing Uses of COVID-19 Vaccines in the Context of Limited Supply: An Approach to Inform Planning and Subsequent Recommendations Based on Epidemiological Setting and Vaccine Supply Scenarios. https://apps.who.int/iris/handle/10665/342917.

[4] Neumann-Böhme S, Varghese NE, Sabat I (2020). Once we have it, will we use it? A European survey on willingness to be vaccinated against COVID-19. Eur J Health Econ.

[5] Althaqafi A, Munshi A, Mujalled MK (2023). COVID-19 vaccine knowledge and attitude among healthcare workers in Jeddah, Saudi Arabia. Cureus.

[6] Sharun K, Rahman F, Haritha V, Jose B, Tiwari R, Dhama K (2020). COVID-19 vaccine acceptance: beliefs and barriers associated with vaccination among the general population in India. J Experiment Biol Agricult Sci.

[7] Izda V, Jeffries MA, Sawalha AH (2021). COVID-19: a review of therapeutic strategies and vaccine candidates. Clin Immunol.

[8] (2021). General Authority for Statistics, Kingdom of Saudi Arabia. https://www.stats.gov.sa/en/node.

[9] Lazarus JV, Ratzan SC, Palayew A (2021). A global survey of potential acceptance of a COVID-19 vaccine. Nat Med.

[10] Alqudeimat Y, Alenezi D, AlHajri B (2021). Acceptance of a COVID-19 vaccine and its related determinants among the general adult population in Kuwait. Med Princ Pract.

[11] Almaghaslah D, Alsayari A, Kandasamy G, Vasudevan R (2021). COVID-19 vaccine hesitancy among young adults in Saudi Arabia: a cross-sectional web-based study. Vaccines (Basel).

[12] Alfageeh EI, Alshareef N, Angawi K, Alhazmi F, Chirwa GC (2021). Acceptability of a COVID-19 vaccine among the Saudi population. Vaccines (Basel).

[13] El-Elimat T, AbuAlSamen MM, Almomani BA, Al-Sawalha NA, Alali FQ (2021). Acceptance and attitudes toward COVID-19 vaccines: a cross-sectional study from Jordan. PLoS One.

[14] Sallam M (2021). COVID-19 vaccine hesitancy worldwide: a concise systematic review of vaccine acceptance rates. Vaccines (Basel).

[15] Paul E, Steptoe A, Fancourt D (2021). Attitudes towards vaccines and intention to vaccinate against COVID-19: implications for public health communications. Lancet Reg Health Eur.

[16] (2021). CDC. Myths and facts about COVID-19 vaccines. https://www.cdc.gov/coronavirus/2019-ncov/vaccines/facts.html.

[17] Brislin RW (1970). Back-translation for cross-cultural research. J Cross Cult Psychol.

[18] Yancy CW (2020). COVID-19 and African Americans. JAMA.

[19] Al-Mohaithef M, Padhi BK (2020). Determinants of COVID-19 vaccine acceptance in Saudi Arabia: a web-based national survey. J Multidiscip Healthc.

[20] Abebe H, Shitu S, Mose A (2021). Understanding of COVID-19 vaccine knowledge, attitude, acceptance, and determinates of COVID-19 vaccine acceptance among adult population in Ethiopia. Infect Drug Resist.

[21] Al-Marshoudi S, Al-Balushi H, Al-Wahaibi A (2021). Knowledge, attitudes, and practices (KAP) toward the COVID-19 vaccine in Oman: a pre-campaign cross-sectional study. Vaccines (Basel).

[22] Islam MS, Siddique AB, Akter R, Tasnim R, Sujan MS, Ward PR, Sikder MT (2021). Knowledge, attitudes and perceptions towards COVID-19 vaccinations: a cross-sectional community survey in Bangladesh. BMC Public Health.

[23] Rahman MM, Chisty MA, Alam MA (2022). Knowledge, attitude, and hesitancy towards COVID-19 vaccine among university students of Bangladesh. PLoS One.

[24] Abu Hammour K, Abu Farha R, Manaseer Q, Al-Manaseer B (2022). Factors affecting the public's knowledge about COVID-19 vaccines and the influence of knowledge on their decision to get vaccinated. J Am Pharm Assoc (2003).

[25] Chilongola JO, Rwegoshola K, Balingumu O, Semvua HS, Kwigizile ET (2022). COVID-19 knowledge, attitudes, practices, and vaccination hesitancy in Moshi, Kilimanjaro region, Northern Tanzania. Tanzania J Health Res.

[26] Kibi S, Shaholli D, Barletta VI (2023). Knowledge, attitude, and behavior toward COVID-19 vaccination in young Italians. Vaccines (Basel).

